# Online calculators for predicting the risk of anastomotic stricture after hepaticojejunostomy for bile duct injury after cholecystectomy: a multicenter retrospective study

**DOI:** 10.1097/JS9.0000000000000404

**Published:** 2023-04-18

**Authors:** Jiangming Chen, Zixiang Chen, Xiyang Yan, Xiaoliang Liu, Debao Fang, Xiang Miao, Zhong Tong, Xiaoming Wang, Zheng Lu, Hui Hou, Cheng Wang, Xiaoping Geng, Fubao Liu

**Affiliations:** a Department of General Surgery, the First Affiliated Hospital of Anhui Medical University; b Department of General Surgery, the Second Affiliated Hospital of Anhui Medical University; c Department of General Surgery, the Fourth Affiliated Hospital of Anhui Medical University; d Department of General Surgery, Anqing Municipal Hospital of Anhui Medical University; e Department of General Surgery, the Third Affiliated Hospital of Anhui Medical University; f Department of General Surgery, the First Affiliated Hospital of Wannan Medical College; g Department of General Surgery, the First Affiliated Hospital of Bengbu Medical College; h Department of General Surgery, the First Affiliated Hospital of University of Science and Technology, Hefei, Anhui Province, China

**Keywords:** anastomotic stricture, bile duct injury, hepaticojejunostomy, prediction

## Abstract

Anastomotic stricture is a common underlying cause of long-term morbidity after hepaticojejunostomy (HJ) for bile duct injury (BDI) following cholecystectomy. However, there are no methods for predicting stricture risk. This study was aimed at establishing two online calculators for predicting anastomotic stricture occurrence (ASO) and stricture-free survival (SFS) in this patient population.

**Methods:**

The clinicopathological characteristics and follow-up information of patients who underwent HJ for BDI after cholecystectomy from a multi-institutional database were reviewed. Univariate and multivariate analyses of the risk factors of ASO and SFS were performed in the training cohort. Two nomogram-based online calculators were developed and validated by internal bootstrapping resamples (*n*=1000) and an external cohort.

**Results:**

Among 220 screened patients, 41 (18.64%) experienced anastomotic strictures after a median follow-up of 110.7 months. Using multivariate analysis, four variables, including previous repair, sepsis, HJ phase, and bile duct fistula, were identified as independent risk factors associated with both ASO and SFS. Two nomogram models and their corresponding online calculators were subsequently developed. In the training cohort, the novel calculators achieved concordance indices (*C*-indices) of 0.841 and 0.763 in predicting ASO and SFS, respectively, much higher than those of the above variables. The predictive accuracy of the resulting models was also good in the internal (*C*-indices: 0.867 and 0.821) and external (*C*-indices: 0.852 and 0.823) validation cohorts.

**Conclusions:**

The two easy-to-use online calculators demonstrated optimal predictive performance for identifying patients at high risk for ASO and with dismal SFS. The estimation of individual risks will help guide decision-making and long-term personalized surveillance.

## Introduction

Highlights

- The first model for predicting the stricture risk of patients who underwent hepaticojejunostomy (HJ) for bile duct injury (BDI) after cholecystectomy.
- Our model demonstrated optimal predictive performance for identifying patients at high risk.
- This easy-to-use online calculator can be used by both clinicians and patients.
- Estimating individual risks will help guide decision-making and long-term personalized surveillance.

BDI remains a potentially devastating complication following cholecystectomy, with a possible reduced life quality and a high rate of subsequent litigation. The treatment of BDI may vary from simple stenting of minor injuries to complicated surgical reconstruction in the case of major injuries. Roux-en-Y HJ is the preferred method for severe BDI, and a series of studies have shown good outcomes after this procedure^1^–^5^.

Although advances in surgical techniques and perioperative management have made HJ a safe procedure for BDI over the past decades, anastomotic stricture, a major complication after HJ and the leading cause of long-term major morbidity, remains a major concern. The incidence of anastomotic stricture reportedly ranges from 10 to 30%^6^,^7^. Patients who develop anastomotic strictures also experience inevitable readmission, increased medical costs, and prolonged psychological pressure. Accordingly, the accurate prediction of stricture can help surgeons identify high-risk patients and perform timely interventions. To the best of our knowledge, however, limited number of studies have reported the factors associated with long-term prognosis after HJ, and no predictive models have been proposed for either anastomotic stricture occurrence (ASO) or stricture-free survival (SFS). As such, it is desirable to develop an accurate and pragmatic model for predicting ASO and SFS.

In this study, we sought to identify the parameters independently influencing ASO and SFS and incorporated them into two nomogram-based calculators to assess the risk for anastomotic stricture in patients undergoing HJ for BDI after cholecystectomy to facilitate the calculation of the risk/benefit ratio of individual patients to guide decision-making.

## Methods

### Study population

Using a multicenter database, with approval from the institutional review boards of each participating institution, data on consecutive patients who underwent HJ for IBDI after cholecystectomy were identified between 2002 and 2022 in the eight hepato-pancreatic-biliary centers of China. The exclusion criteria were as follows: (1) perioperative death; (2) missing data on important clinical or follow-up variables. All HJ procedures of patients enrolled in this study were performed in an open approach. BDI was defined as any damage to the bile duct or biliary tree including bile leaks^8^. All the patients in this study were with major BDI. The type of BDI was defined by Strasberg classification, in which type E1 is a circumferential injury to the common duct more than 2 cm from the bifurcation; type E2 is a circumferential injury to the common duct less than 2 cm from the bifurcation; type E3 is a circumferential injury to the common duct at the bifurcation; type E4 is injury proximal to the bifurcation involving both main right and left hepatic ducts; and type E5 is a combined injury to the common duct and a major aberrant right hepatic duct^9^,^10^. This retrospective study was conducted in accordance with the Declaration of Helsinki and the Ethical Guidelines for Clinical Studies (No. Quick-PJ 2022-08-29) and was considered exempt from requiring informed consent. Besides, this study was registered with ResearchRegistry.com (Unique Identification Number: researchregistry8389). Data have been reported as per STROCSS 2021 criteria^11^.

All postcholecystectomy BDI repair was performed via Roux-en-Y HJ by specialized hepatobiliary surgeons from the participating centers. Dissection and adhesiolysis were started towards the liver hilum to identify and lengthen the healthy bile duct stump. Division of hilar plate and limited hepatectomy were performed when needed. The common hepatic, left and right hepatic ducts were further dissected based on BDI level until the healthy and vascularized biliary stump was reached. The segmental ducts were sutured together, if possible, to enable the construction in one, rather than two jejunal anastomoses. A 40–60 cm long Roux-en-Y jejunal loop was prepared and then transferred to the right abdominal cavity through the transverse mesocolon on the right edge of the median colonic pedicle. HJ was performed as wide as possible by end-to-side, mucosa-mucosa, single-layer of Vicryl, or polydioxanone synthetic (4/0 or 5/0) sutures (interrupted, continuous, or both). At least one intra-abdominal drain was placed behind the anastomosis. The diameter of the proximal bile duct used for HJ after BDI was generally unchanged or dilated compared with that before cholecystectomy. Therefore, a stent was not routinely used during HJ, and no such patients were enrolled in this study. No technical modifications were made during the study period.

### Follow-up and data collection

Patients were followed up after 1 month, 3 months, 6 months, 1 year, and then annually thereafter. Each visit included a physical examination, liver function test, and abdominal ultrasound to determine the status of the liver. Magnetic resonance cholangiopancreatography was performed in patients presenting with recurrent cholangitis to assess the patency of HJ. Telephone interviews were used as a complementary follow-up method. Baseline data, clinical features, intraoperative variables, hospital morbidity and mortality, and long-term prognosis were recorded. Previous repair in this study was defined as any attempt at biliary repair between BDI and specialist HJ after referral^12^, such as primary suture or clip, repair over T-tube, HJ, and end-to-end anastomosis. The HJ phase was defined as early (<14 days), intermediate (14–90 days), or late (>90 days after BDI). Sepsis was assessed in the presence of at least one of the following features: leukocytosis more than 15 000/ml, fever >38.5°C, angiocholitis episodes, peritonitis, or intra-abdominal abscess^13^. Bile fistula was defined as bilirubin concentration in the drains exceeding serum bilirubin with a consecutive change of clinical management or occurrence of a bilioma necessitating drainage^14^. All data were screened and collected from the computerized BDI database by a specialized research assistant.

### Predictive endpoints

As the present study focused on post-HJ anastomotic strictures, the predictive endpoints were ASO and SFS. Anastomotic stricture was defined as the presence of abdominal symptoms, cholangitis, and abnormal liver function tests in conjunction with a stricture at the HJ, diagnosed with percutaneous transhepatic cholangiography, computed tomography, or MRI, requiring intervention^15^. SFS was calculated from the date of HJ to either the date of stricture occurrence or the date of the last follow-up.

### Statistical analysis

Categorical variables were summarized as numbers (*n*) and proportions (%) and compared using χ^2^ test or Fisher’s exact test, as appropriate. The survival curve of SFS was manifested using Kaplan–Meier method and compared using the log-rank test. Logistic and Cox proportional hazards regression models were used to identify independent factors for predicting ASO and SFS, respectively. Variables with significance (*P*<0.10 in the univariate logistic and Cox regression analyses) were included in the subsequent multivariate regression analysis.

Two calculators for ASO and SFS were generated independently to predict individual risks based on the significant factors originating from the multivariate analysis performed by a backward step-down selection process applying a threshold of *P*-value less than 0.05. The performance of the models was evaluated visually using Harrell’s *C*-index and calibration curves by comparing the nomogram predictions with the actual observed endpoints^16^.

For validation, the discrimination and calibration capabilities of the calculators from the training cohort were verified using the same methods as in the external validation cohort. The bootstrapping technique (1000 repetitions), which is based on random sampling with replacement, was used for internal validation. In addition, the predictive performances of the two models and any of the four screened factors were compared using receiver operating characteristic (ROC) curves and decision curve analysis (DCA)^17^,^18^. To facilitate their incorporation into clinical practice, the model formulas were also coded into web-compatible versions. Statistical analyses were conducted using IBM SPSS Statistic, version 23.0 (IBM Corp.), and R software version 4.1.3 (http://www.r-project.org/). A *P*-value less than 0.05 was considered to indicate a significant difference in a two-tailed test.

## Results

### Patient cohorts and clinicopathologic features

**Figure 1:**
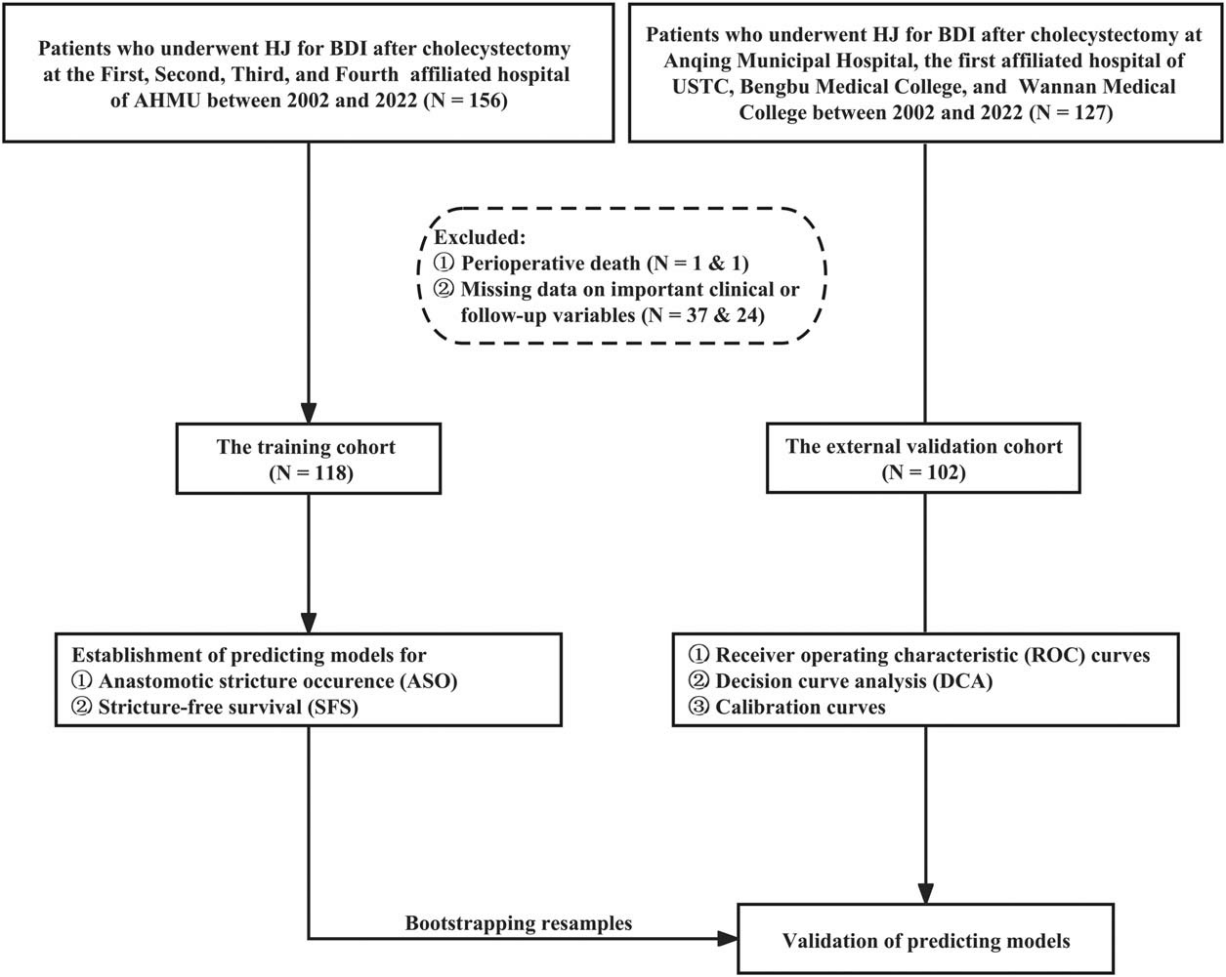
Flowchart of patient enrollment. AHMU, Anhui Medical University; BDI, bile duct injury; HJ, hepaticojejunostomy; USTC, University of Science and Technology of China.

During the study period, 283 patients who underwent HJ for BDI after cholecystectomy at eight hepato-pancreatic-biliary centers. Among them, 63 (22.26%) patients who met the exclusion criteria were excluded: 2 (0.71%) died in the perioperative period and 61 (21.55%) lacked complete clinical or follow-up data. A total of 220 patients were enrolled in the whole cohort, including 51 (23.18%) males and 169 (76.82%) females. Specifically, 118 (46.09%) patients from the First, Second, Third, and Fourth affiliated hospitals of Anhui Medical University were identified and set as the training cohort. Based on the same screening criteria, an independent group consisting of 102 (36.04%) patients from the remaining four centers during the same period were included in the present study and served as an external validation cohort (Fig. 1). Attempts to repair BDI before specialist HJ were performed in 59 (26.82%) patients in the whole cohort, including primary suture or clip [19 (32.20%)], repair over T-tube [16 (27.12%)], HJ [13 (22.03%)], and end-to-end anastomosis [11 (18.64%)]. There were no significant differences (*P*<0.05) in baseline characteristics between the two cohorts as shown in Table 1.

**Table 1:** Comparisons of clinicopathological characteristics and operative variables between the training and validation cohorts

	*n* (%)			
Clinical variables	Whole cohort (*N*=220)	Training cohort (*n*=118)	Validation cohort (*n*=102)	*P*
Sex				
Male	51 (23.18)	25 (21.19)	26 (25.49)	0.552
Female	169 (76.82)	93 (78.81)	76 (74.51)	
Age (years)				
≤60	135 (61.36)	70 (59.32)	65 (63.73)	0.596
>60	85 (38.64)	48 (40.68)	37 (36.27)	
Sepsis				
No	178 (80.91)	99 (83.90)	79 (77.45)	0.298
Yes	42 (19.09)	19 (16.10)	23 (22.55)	
Strasberg grade				
E1–E2	97 (44.09)	47 (39.83)	50 (49.02)	0.218
E3–E5	123 (55.91)	71 (60.17)	52 (50.98)	
Transfer timing (days)				
≤14	101 (45.91)	55 (46.61)	46 (45.10)	0.929
>14	119 (54.09)	63 (53.39)	56 (54.90)	
TBIL (μmol/l)				
≤34.2	89 (40.45)	45 (38.14)	44 (43.14)	0.538
>34.2	131 (59.55)	73 (61.86)	58 (56.86)	
Pre-HJ repair				
No	161 (73.18)	80 (67.80)	81 (79.41)	0.052
Yes	59 (26.82)	38 (32.20)	21 (20.59)	
Pre-HJ drainage				
No	142 (64.55)	82 (69.49)	60 (58.82)	0.132
Yes	78 (35.45)	36 (30.51)	42 (41.18)	
Bile duct diameter				
Unchanged	96 (43.64)	49 (41.53)	47 (46.08)	0.586
Dilated	124 (56.36)	69 (58.47)	55 (53.92)	
Suture method				
Interrupted	115 (52.27)	59 (50.00)	56 (54.90)	0.757
Continuous	83 (37.73)	47 (39.83)	36 (35.29)	
Both	22 (10.00)	12 (10.17)	10 (9.80)	
HJ timing (days)				
≤14	58 (26.36)	34 (28.81)	24 (23.53)	0.669
14–90	47 (21.36)	24 (20.34)	23 (22.55)	
>90	115 (52.27)	60 (50.85)	55 (53.92)	
Bile duct stone when HJ				
No	182 (82.73)	95 (80.51)	87 (85.29)	0.449
Yes	38 (17.27)	23 (19.49)	15 (14.71)	
Hepatectomy				
No	206 (93.64)	111 (94.07)	95 (93.14)	0.996
Yes	14 (6.36)	7 (5.93)	7 (6.86)	
Operation duration (min)				
≤180	159 (72.27)	86 (72.88)	73 (71.57)	0.947
>180	61 (27.73)	32 (27.12)	29 (28.43)	
Bile fistula				
No	162 (73.64)	85 (72.03)	77 (75.49)	0.670
Yes	58 (26.36)	33 (27.97)	25 (24.51)	
Postoperative stay (days)				
≤15	152 (69.09)	87 (73.73)	65 (63.73)	0.146
>15	68 (30.91)	31 (26.27)	37 (36.27)	
Intraoperative hemorrhage (ml)				
≤400	195 (88.64)	100 (84.75)	95 (93.14)	0.081
>400	25 (11.36)	18 (15.25)	7 (6.86)	
Stricture				
No	179 (81.36)	97 (82.20)	82 (80.39)	0.865
Yes	41 (18.64)	21 (17.80)	20 (19.61)	

HJ, hepaticojejunostomy; TBIL, total bilirubin.

### Postoperative short-term and long-term outcomes

**Figure 2:**
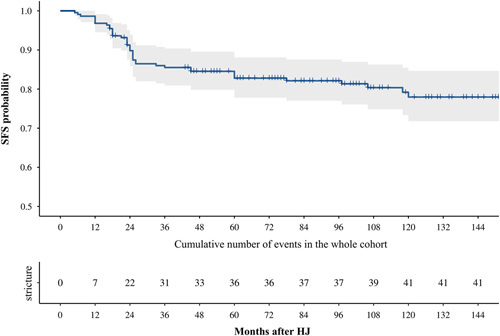
Kaplan–Meier curves for SFS of patients after HJ for bile duct injury in the whole cohort. HJ, hepaticojejunostomy; SFS, stricture-free survival.

Among the 118 patients in the training cohort, 19 (16.10%) suffered major morbidities (Clavien–Dindo grade III–V)^19^ within 30 days after HJ, including 33 (27.97%) with bile fistula; 2 (1.69%) with hemorrhage; and 4 (3.39%) with abdominal infections. Accordingly, major morbidities, including 25 (24.51%) with bile fistula, 2 (1.96%) with hemorrhage, and 3 (2.94%) with abdominal infections were observed in the validation cohort. Regarding the long-term prognosis, at the final follow-up, 21 (18.64%) patients in the training cohort had developed ASO with a median SFS of 101.0 months (interquartile range, 93.2–108.8 months). The 2-year, 5-year, and 10-year SFS rates were 89.5, 81.9, and 80.4%, respectively. In the validation cohort, 20 (19.61%) patients had developed ASO with a median SFS of 105.0 months (interquartile range, 93.5–116.5 months). The 2-year, 5-year, and 10-year SFS rates were 90.1, 83.7, and 75.4%, respectively. More than 50 and 75% of the ASOs were found within 24 months (22/41) and 36 months (31/41) after HJ in the whole cohort, respectively. The downward trend of the Kaplan–Meier curve became more moderate as the follow-up duration extended (Fig. 2). Thirty-six patients (87.80%) with ASO were treated with surgical anastomotic reconstruction, followed by 2 (4.88%) with endoscopic retrograde cholangiopancreatography, and 3 (7.32%) with percutaneous transhepatic choledochoscopic lithotomy in the whole cohort (Table 2).

**Table 2:** Comparisons of short-term (postoperative 30 days) and long-term outcomes between the training and validation cohorts

	*n* (%)			
Clinical variables	Whole cohort (*N*=220)	Training cohort (*n*=118)	Validation cohort (*n*=102)	*P*
Short-term outcomes				
Bile fistula				
No	162 (73.64)	85 (72.03)	77 (75.49)	0.670
Yes	58 (26.36)	33 (27.97)	25 (24.51)	
Postoperative hemorrhage				
No	216 (98.18)	116 (98.31)	100 (98.04)	0.883
Yes	4 (1.86)	2 (1.69)	2 (1.96)	
Abdominal infection				
No	213 (96.82)	114 (96.61)	99 (97.06)	0.850
Yes	7 (3.18)	4 (3.39)	3 (2.94)	
Major morbidity				
No	183 (83.18)	99 (83.90)	84 (82.35)	0.760
Yes	37 (16.82)	19 (16.10)	18 (17.65)	
Long-term outcomes				
Stricture				
No	179 (81.36)	97 (82.20)	82 (80.39)	0.865
Yes	41 (18.64)	21 (17.80)	20 (19.61)	
Treatment of stricture				
Reconstruction	36 (87.80)	18 (85.71)	18 (90.00)	0.999
ERCP	2 (4.88)	1 (4.76)	1 (5.00)	
PTCSL	3 (7.32)	2 (9.52)	1 (5.00)	
SFS [median (95% CI)] (months)	103.0 (97.8–108.2)	101.0 (93.2–108.8)	105.0 (93.5–116.5)	0.823
2-year SFS rate (%)	89.8	89.5	90.1	
5-year SFS rate (%)	82.8	81.9	83.7	
10-year SFS rate (%)	78.0	80.4	75.4	

ERCP, endoscopic retrograde cholangiopancreatography; PTCSL, percutaneous transhepatic choledochoscopic lithotomy; SFS, stricture-free survival.

### Development of online calculators

**Figure 3:**
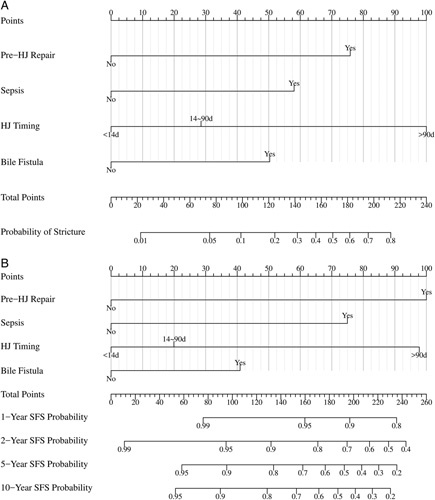
The nomogram models for predicting anastomotic stricture occurrence (A) and SFS (B) for patients after HJ for bile duct injury. HJ, hepaticojejunostomy; SFS, stricture-free survival.

**Figure 4:**
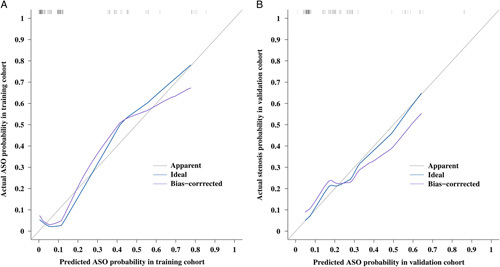
Calibration plots of the model for predicting anastomotic stricture occurrence (ASO) in the training (A) and validation (B) cohorts.

**Figure 5:**
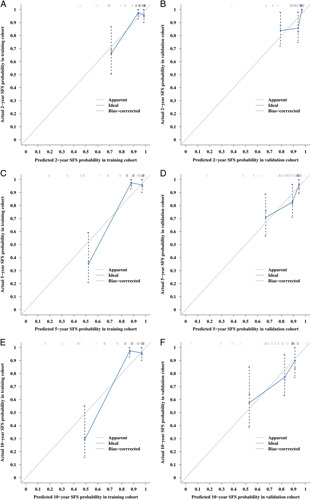
Calibration plots of the model for predicting 2-year, 5-year, and 10-year stricture-free survival (SFS) in the training (A, C and E) and validation (B, D and F) cohorts.

**Figure 6:**
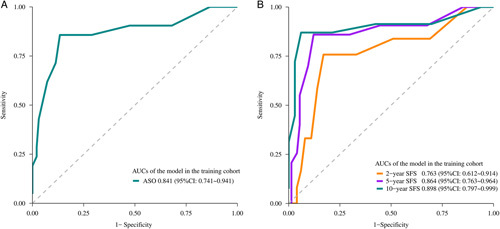
Receiver operating characteristic curves of the models for predicting ASO (A), 2-year, 5-year, and 10-year SFS (B). ASO, anastomotic stricture occurrence; AUC, area under the curve; SFS, stricture-free survival.

The results of univariate and multivariate analyses of ASO and SFS based on accessible variables in the training cohort are listed in Tables 3 and 4, respectively. Factors that significantly affected ASO and SFS in the univariate analysis were subjected to multivariate analysis. Finally, four variables, including previous repair, sepsis, HJ phase, and bile duct fistula, which were identified as independent risk factors associated with both ASO and SFS from the multivariate analysis (*P*<0.05), were used to construct the nomograms (Fig. 3). Both models demonstrated satisfactory precision in predicting stricture risk. In the training cohort, the *C*-indices were 0.841 (95% CI: 0.703–0.873) for ASO and 0.763 (95% CI: 0.708–0.782) for SFS. The performance was well validated in the internal bootstrapping resamples (*C*-indices: ASO: 0.867, 95% CI: 0.757–0.948; SFS: 0.821, 95% CI: 0.704–0.918), as well as the external validation cohort (*C*-indices: ASO: 0.852, 95% CI: 0.792–0.862; SFS: 0.823, 95% CI: 0.775–0.869). Two nomogram-based online calculators were further programed, and the nomograms are freely available at: https://stricture.shinyapps.io/ASO_model/ for ASO and https://stricture.shinyapps.io/SFS_model/ for SFS. Satisfied calibration curves were also obtained for risk estimation in the training and validation cohorts (Figs. 4 and 5). In addition, ROC curves for the two calculators were plotted in the training cohort (Fig. 6).

**Table 3:** Univariate and multivariate logistic regression analyses for preoperative predicting stricture occurrence in the training cohort

Variables	OR comparison	UV [OR (95% CI)]	UV (*P*)	MV [OR (95% CI)]	MV (*P*)^a^
Sex	Female vs. male	0.46 (0.16–1.29)	0.139		
Age	>60 vs. ≤60 years	1.78 (0.69–4.61)	0.232		
Sepsis	Yes vs. no	6.52 (2.21–19.29)	0.001^a^	5.46 (1.31–26.18)	**0.024**
Strasberg grade	E3–E5 vs. E1–E2	0.54 (0.21–1.39)	0.199		
Transfer timing	>14 vs. ≤14	0.60 (0.23–1.55)	0.289		
TBIL	>34.2 vs. ≤34.2 μmol/l	1.29 (0.48–3.48)	0.618		
Pre-HJ repair	Yes vs. no	4.68 (1.74–12.61)	0.002^a^	12.09 (2.67–76.12)	**0.003**
Pre-HJ drainage	Yes vs. no	0.67 (0.22–1.98)	0.464		
Bile duct diameter	Dilated vs. unchanged	1.53 (0.57–4.12)	0.403		
Suture method	Interrupted vs. both	0.90 (0.17–4.81)	0.902		
	Continuous vs. both	1.35 (0.25–7.19)	0.724		
HJ timing	14–90 vs. <4 days	4.60 (0.56–38.09)	0.157	2.26 (0.21–69.22)	0.07
	>90 vs. <14 days	9.58 (1.14–80.91)	0.038^a^	15.3 (1.26–60.13)	**0.031**
Bile duct stone when HJ	Yes vs. no	1.37 (0.44–4.23)	0.583		
Hepatectomy	Yes vs. no	1.76 (0.56–2.21)	0.991		
Operation duration	>180 vs. ≤180 min	5.13 (1.90–13.88)	0.001^a^	3.18 (0.78–13.66)	0.106
Bile fistula	Yes vs. no	3.75 (1.41–9.99)	0.008^a^	5.68 (1.59–22.28)	**0.009**
Postoperative stay	>15 vs. ≤15 days	0.85 (0.28–2.56)	0.778		
Intraoperative hemorrhage	>400 vs. ≤400 ml	2.83 (0.92–8.71)	0.069^a^	0.68 (0.12–3.45)	0.649

HJ, hepaticojejunostomy; MV, multivariate; OR, odds ratio; TBIL, total bilirubin; UV, univariate.

a Variables found significant at *P*-value less than or equal to 0.1 in univariate analysis were entered into multivariate analysis.

Variables found significant at *P* ≤ 0.05 in multivariate analyses were marked in bold.

**Table 4:** Univariate and multivariate Cox regression analyses for preoperative predicting stricture-free survival in the training cohort

Variables	HR comparison	UV [HR (95% CI)]	UV (*P*)	MV [HR (95% CI)]	MV (*P*)^a^
Sex	Female vs. male	0.55 (0.22–1.38)	0.203		
Age	>60 vs. ≤60 years	1.57 (0.67–3.71)	0.299		
Sepsis	Yes vs. no	5.39 (2.26–12.87)	<0.001^a^	4.08 (1.45–11.48)	**0.008**
Strasberg grade	E3–E5 *vs* E1–E2	0.62 (0.27–1.47)	0.282		
Transfer timing	>14 vs. ≤14	0.55 (0.23–1.3)	0.174		
TBIL	>34.2 vs. ≤34.2 μmol/l	1.32 (0.53–3.28)	0.545		
Pre-HJ repair	Yes vs. no	4.35 (1.8–10.53)	0.001^a^	9.92 (2.64–37.21)	**0.001**
Pre-HJ drainage	Yes vs. no	0.64 (0.23–1.74)	0.382		
Bile duct diameter	Dilated vs. unchanged	1.56 (0.63–3.86)	0.340		
Suture method	Interrupted vs. both	0.91 (0.20–4.20)	0.901		
	Continuous vs. both	1.39 (0.30–6.32)	0.674		
HJ timing	14–90 vs. <14 days	4.49 (0.57–35.1)	0.152	1.47 (0.17–12.49)	0.084
	>90 vs. <14 days	7.94 (1.02–62.11)	0.048^a^	6.72 (1.76–58.44)	**0.013**
Bile duct stone when HJ	Yes vs. no	1.41 (0.52–3.85)	0.502		
Hepatectomy	Yes vs. no	2.11 (0.68–3.74)	0.997		
Operation duration	>180 vs. ≤180 min	3.67 (1.55–8.72)	0.003^a^	1.34 (0.48–3.73)	0.571
Bile fistula	Yes vs. no	3.00 (1.27–7.06)	0.012^a^	2.79 (1.02–7.66)	**0.047**
Postoperative stay	>15 vs. ≤15 days	0.78 (0.29–2.14)	0.637		
Intraoperative hemorrhage	>400 vs. ≤400 ml	2.65 (1.03–6.85)	0.044^a^	0.50 (0.15–1.71)	0.267

HJ, hepaticojejunostomy; HR, hazard ratio; MV, multivariate; TBIL, total bilirubin; UV, univariate.

a Variables found significant at *P*-value less than or equal to 0.1 in univariate analysis were entered into multivariate analysis.

Variables found significant at *P* ≤ 0.05 in multivariate analyses were marked in bold.

### Comparisons of the models with four independent predictors

**Figure 7:**
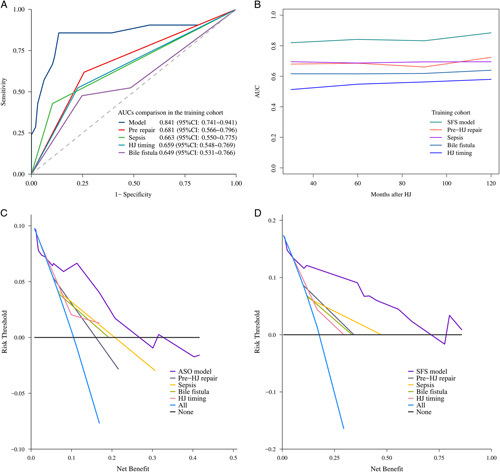
Receiver operating characteristic curves and decision curve analysis of the models for predicting stenosis occurrence (A and C), SFS (B and D) in the training cohort. ASO, anastomotic stricture occurrence; AUC, area under the curve; HJ, hepaticojejunostomy; SFS, stricture-free survival.

Comparisons of the discriminatory performance and predictive power between the calculators and the four independent predictors of ASO and SFS using ROC and DCA curves are shown in Figure 7. The areas under the curve of the proposed models in the training cohort were superior to those of any of the four variables in predicting ASO (area under the curve: 0.841 vs. 0.649–0.681) and SFS (time-dependent ROC). Furthermore, Figure 7 also demonstrates that the proposed models had the highest DCAs over most ranges of the probability thresholds, suggesting that the two models were more beneficial than independent factors and the best ability to predict the two study endpoints.

## Discussion

Despite the popularity of cholecystectomy, BDI remains a severe complication related to cholecystectomy and may result in both immediate and long-term morbidity and mortality. Patients with severe BDI may require surgical intervention. A retrospective study of a nationwide dataset of patients who underwent cholecystectomy between 2001 and 2011 demonstrated that the incidence of BDI requiring surgical repair was 0.1%^20^. The long-term prognosis of repair for BDI can be dismal even in high-volume hepatobiliary centers with experienced surgeons. The stricture rate after HJ ranged from 10 to 30%^7^. The stricture rate in the present study was 18.64%, with most of the strictures occurring in the second year, and the median interval between HJ and stricture was 34.34 months, which is consistent with the findings of Stilling *et al.* 7. Therefore, the high incidence of stricture after HJ is a major risk factor associated with the long-term life quality of patients with BDI.

Accurate prediction of ASO and SFS can potentially help implement individualized therapy for patients undergoing HJ for BDI after cholecystectomy. Although multiple efforts have been made to explore the association between clinical variables and long-term stricture situations, none of these studies have further developed a prediction model^12^,^15^,^21^. In this scenario, the present study, therefore, developed and externally validated two nomogram-based calculators that could accurately predict ASO and SFS in this population. By considering a wide variety of prognostic factors and complex mathematical relationships, the current calculators individualized the risk for ASO and SFS for each patient and demonstrated a greater prognostic significance than the four independent risk factors, including previous repair, sepsis, HJ phase, and bile fistula. These four predictors can be easily accessed, making the models feasible in clinical practice. Furthermore, consistent with our results, previous studies have determined these factors as independent factors for ASO and SFS^12^,^15^,^21^,^22^.

Almost all relevant studies have shown that the timing of sound biliary repair by experienced surgeons is a critical factor in improving the prognosis of BDI patients^5^,^22^–^24^. Previous studies revealed that the success rate of repair performed by specialists can reach 79%; however, it is lower than 30% in the case of nonspecialists or beginners^25^,^26^. We concluded that nonspecialist repair may exert adverse effects on the outcomes of BDI in both psychological and technical aspects. First, nonspecialists tend to minimize the subsequent influence of intraoperative adverse incidents and are timid in the treatment of BDI. Second, limited by experience, nonspecialists are not likely to make a comprehensive assessment of the degree of BDI and thus, an appropriate treatment would not be chosen. Third, nonspecialists are inferior to specialists in the selection of anastomotic techniques, anastomotic materials, and management of postoperative complications.

Repair timing was classified into early, intermediate, and late phases. The traditional view is that repair surgery should be performed 3 months after biliary duct injury to facilitate HJ when the biliary wall and surrounding inflammation completely subside, the proximal bile duct dilates, and the range of bile duct ischemia is clear. With the development of surgical skills and equipment, emerging evidence supports the safety of early repair at experienced centers. Particularly, proven skills for thin bile duct anastomosis used in liver transplantation also bring about more probabilities for early repair. Perera et al. compared the outcomes of two groups of patients with BDI and found that immediate and early repair after BDI results in comparable, if not better, long-term outcomes compared with late repair when performed by specialists, with stricture rates of 18, 5, and 29%, respectively^27^. This study also suggested that repair attempts in low-volume hospitals before referral and late repair were associated with a higher risk for ASO and SFS. Besides, the suture method was not an independent risk factor associated with both ASO and SFS, which suggested that it was the quality rather than the method of anastomosis that independently affected the prognosis of patients with BDI. As such, a direct repair by experienced surgeons in the early phase in high-volume hospitals was highly recommended.

A large cohort study of 529 BDI patients who underwent HJ revealed that sepsis control before the repair was a protective factor against anastomotic failure, suggesting that adequate antibiotic and drainage treatment should be considered for patients with sepsis^28^. Bile duct fistula after HJ was also shown to increase the rate of anastomotic stricture after HJ, and bile fistula may induce a pre-anastomotic inflammatory response that resulted in fibrosis with stricture formation. In addition, based on our institutional experience, the placement of two drainages above and below the anastomotic stoma respectively during HJ for adequate drainage and removing them successively are beneficial for the management of bile fistula. More than 50% of the ASOs were diagnosed within 24 months and 75% within 36 months. These earlier ASOs were associated more with perioperative factors, including the four predictors and surgical techniques, while latter ASOs were associated more with the anatomical changes of the digestive system and nonfunctioning of Oddi sphincter after HJ^29^,^30^. The trend revealed by the Kaplan–Meier curve was also consistent with that in previous studies^7^,^15^,^31^–^33^. Therefore, timely interventions regarding these four risk factors can reduce the incidence of ASO and improve the overall prognosis of patients after HJ.

In the present study, we also programmed two online calculators that can be accessed using computers, smartphones, or other mobile devices on the basis of nomograms that significantly improved the approachability of such predictive models. Our predictive model had reasonable discriminatory power with *C*-indices of 0.841 and 0.763 for ASO and SFS, respectively. In addition, the calibration plot demonstrated the satisfactory accuracy of our model in both the training and validation cohorts. In addition, both DCAs and ROCs revealed that the proposed models were the highest over most ranges of probability thresholds, indicating that the models were quite robust. These models provide easy-to-use tools for comprehensive prognosis evaluation and decision-making. For those patients with a high risk for ASO or dismal SFS according to the calculators, perioperative management should be enforced, and follow-up during the first 3 years after HJ should be performed more closely.

The present study has several limitations. First, the retrospective nature of the methodology, institutional variation in surgical practices, and a high proportion of patients lost to follow-up may lead to inherent biases. Second, the models were established based on perioperative data, which made preoperative prediction impossible. In addition, prospective studies are required to validate the reliability of these models.

Irrespective of these limitations, our study has a number of strengths, such as the large sample size, the multi-institutional nature of data, and the use of an external validation dataset. The greatest strength may lie in the fact that this pioneering study provides a comprehensive insight into anastomotic stricture and makes it possible to create a ‘risk flag’ in the electronic medical record by identifying individuals who are at high risk for ASO and poor SFS and then provide individualized management. The potential for the seamless integration of high-value indicators of risk and clinical practice has a broad application prospect, which is also a promising direction for future research.

## Conclusions

The two calculators developed for the first time for predicting the risk for ASO and SFS in patients who underwent HJ for BDI after cholecystectomy showed superior performance and discriminative power compared with those of a single independent factor. Both clinicians and patients can use them to stratify patients according to the underlying risk, allowing the development of personalized surveillance programs and a better distribution of health resources.

## Ethical approval

The study procedures were approved by the Institutional Ethics Committee of the First Affiliated Hospital of Anhui Medical University (No. Quick-PJ 2022-08-29).

## Sources of funding

This study was supported by the University Natural Science Research Project of Anhui Province (No. KJ2021ZD0021). The funding sources had no role in the design and conduct of the study; collection, management, analysis, and interpretation of the data; preparation, review, or approval of the manuscript; and the decision to submit the manuscript for publication.

## Author contribution

J.C.: conceptualization, data curation, formal analysis, writing – original draft. Z.C.: data curation, formal analysis, methodology, software and visualization, writing – original draft. X.Y.: data curation, methodology, software and visualization, writing – original draft. X.L.: data curation, methodology, software, and visualization. D.F., X.M., Z.T., X.W., Z.L., H.H., and C.W.: data curation. X.G.: conceptualization, data curation, methodology, supervision, writing – review and editing. F.L.: conceptualization, formal analysis, funding acquisition, methodology, supervision, writing – original draft, writing – review and editing.

## Conflicts of interest disclosure

The authors declare that they have no financial conflict of interest with regard to the content of this report.

## Research registration unique identifying number (UIN)

1. Name of the registry: Research Registry.
2. Unique Identifying number or registration ID: Researchregistry8389.
3. Hyperlink to your specific registration (must be publicly accessible and will be checked): https://www.researchregistry.com/browse-theregistry#home/registrationdetails/634129e29f48ee0021315fe6/

## Guarantor

Fubao Liu.

## Data availability statement

The data that support the study findings are available upon reasonable request from the corresponding authors (Fubao Liu). The full data are not publicly available due to limitations posed by the ethical regulations at some of the participating centers.
